# Stroke in High‐Altitude Areas

**DOI:** 10.1002/brb3.70626

**Published:** 2025-06-10

**Authors:** Chuanxi Duan, Lijun Wu, Yuding Luo, Jiali Zhang, Pingchuan Liu, Fanzhou Ren, Junhao Li, Hai Xiong, Jian Wang

**Affiliations:** ^1^ North Sichuan Medical College Nanchong China; ^2^ Department of Neurology Ya'an People's Hospital Ya'an China; ^3^ Ya'an Key Laboratory of Plateau Medicine Ya'an China; ^4^ Department of Neurology Fushun People's Hospital Zigong China; ^5^ Department of Neurology, the Affiliated Hospital Southwest Medical University Luzhou China

**Keywords:** flight‐associated stroke, high altitude, post‐stroke cognitive impairment, stroke

## Abstract

**Background:**

Stroke poses a significant social and familial burden on populations worldwide. While research on stroke in low‐altitude areas is extensive, there remains a considerable gap in stroke research in high‐altitude regions.

**Methods:**

A computer‐based search of PubMed and Google Scholar was conducted to retrieve literature on stroke in high‐altitude areas. Cross‐referencing was performed using available articles and other scientific search engines. Relevant studies on stroke in high‐altitude regions were included in this review.

**Results:**

A total of five review articles, two systematic reviews and meta‐analyses, three cross‐sectional studies, nine retrospective clinical studies (including case reports and case‐control studies), one commentary, one letter, and three animal studies were included in this literature review. All data were sourced from high‐altitude regions.

**Conclusion:**

We explored the contribution of environmental and individual factors in high‐altitude areas to the occurrence and progression of stroke, and highlighted the current research advances in ischemic stroke, hemorrhagic stroke, post‐stroke cognitive impairment in high‐altitude regions, and flight‐related stroke. In light of the current limitations in research on stroke in these areas, we propose feasible directions for future studies, aiming to provide insights for future research on stroke in high‐altitude regions.

## Graphical abstract

This study provides a literature review exploring the relationship between high‐altitude environmental factors and stroke, analyzes the limitations of current research, and proposes future research directions and preventive measures.

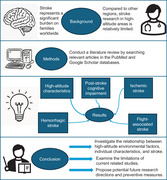

## 1 Introduction

The 2021 Global Burden of Disease Study (GBD) (Murray 2022) covers 371 diseases and injuries, 88 risk factors, and 204 countries and regions, revealing that stroke is one of the leading causes of death and disability worldwide. Stroke is the second leading cause of death and the third leading cause of disability‐adjusted life years (DALYs) among noncommunicable diseases globally, with more than 80% of stroke‐related DALYs occurring in low‐ and middle‐income countries (LMICs). From 1990 to 2021, the global crude incidence and prevalence of stroke increased, primarily due to population aging, while the decline in age‐standardized incidence and prevalence rates globally reflects this trend (Wu and Liu 2024).

The 2021 global survey on populations in high‐altitude areas indicates that approximately 500 million people live at altitudes ≥ 1500 m (Tremblay and Ainslie 2021). On the other hand, an increasing number of people are choosing to visit high‐altitude regions for tourism and recreational activities. Some soldiers, miners, guides, and residents of mountainous areas are also exposed to high‐altitude environments for extended periods. To ensure the safety of these populations, advancing high‐altitude medicine is of utmost importance. Regarding the definition of high‐altitude regions, there are currently two main perspectives. One is a broad definition: high‐altitude regions are those with an altitude > 1500 m. The other is a more specific definition: high‐altitude regions are those with an altitude > 2500 m. These differing definitions arise because some scholars base the classification on the International Society of Mountain Medicine's definition (low‐altitude areas below 1500 m, moderate‐altitude areas between 1500 and 2500 m, high‐altitude areas between 2500 and 3500 m, extreme high‐altitude areas between 3500 and 5800 m, and areas above 5800 m as extreme high‐altitude regions with a higher mortality rate) (Imray et al. 2011), whereas other scholars define it based on physiological responses that occur at altitudes ≥ 1500 m (Yan et al. 2022). Therefore, the data in this review are derived from studies conducted at altitudes ≥ 1500 m.

Due to the unique geographic and climatic factors, stroke in high‐altitude regions presents significant differences compared to stroke in low‐altitude areas, as confirmed by previous studies (Zheng et al. 2023). However, research on stroke in high‐altitude regions is relatively scarce compared to that on low‐altitude regions. Therefore, this article aims to provide a review of past literature and the current research progress on stroke in high‐altitude areas, offering valuable insights for the prevention and clinical treatment of stroke in future high‐altitude region populations.

## 2 Methods

To conduct this review, we performed a literature search in PubMed and Google Scholar using the following search terms/keywords: “Stroke at High Altitude,” “Stroke and High Altitude,” “Cerebral hemorrhage at high altitude,” “Cerebral hemorrhage and High Altitude,” “Subarachnoid hemorrhage at High altitude,” “Subarachnoid hemorrhage and High Altitude,” “Ischemic stroke at High altitude,” and “Ischemic stroke and High altitude.” These search terms yielded 427 citations, and after excluding 26 non‐English articles, the final result was 401 English articles. After identifying relevant studies discussing stroke in high‐altitude regions, we conducted cross‐referencing. We found five review articles (Hameed et al. 2024; Syed et al. 2022; Zhang et al. 2020; Cavalcante and Ormond 2018; Ortíz‐Prado and Dunn 2011), two systematic reviews and meta‐analyses (Zheng et al. 2023; Ortiz‐Prado, Cordovez, et al. 2022), three cross‐sectional studies (Tu et al. 2023; X. Li et al. 2023; Z. Li et al. 2022), nine retrospective clinical studies (Yan et al. 2022; Cui et al. 2014; Mahdavi et al. 2010; L. J. Wei et al. 2021; M. Liu et al. 2021; Lu et al. 2020; L. Wei et al. 2019; Chen et al. 2017, 2018) (including case reports, case‐control studies), one commentary (Ortiz‐Prado, Villafuerte, et al. 2022), one letter (Luo et al. 2023), and three animal studies (H. Zhu et al. 2015, 2013; M. Zhu et al. 2021) that were suitable for this review. Based on the content presented in these articles, we will begin by discussing the characteristics of high‐altitude regions and then explore ischemic stroke (IS), hemorrhagic stroke (HS), post‐stroke cognitive impairment (PSCI), and flight‐related strokes.

## 3 Characteristics of High‐Altitude Regions

High‐altitude regions possess unique geographical and climatic characteristics compared to lowland areas. As altitude increases, atmospheric pressure decreases, leading to a reduction in air density, which in turn causes a decrease in oxygen partial pressure (Palmer 2010). Blood oxygen saturation (SpO_2_), which reflects the degree of oxygen binding to hemoglobin, is a good indicator of hypoxic status in the body. In high‐altitude areas, due to the lower oxygen partial pressure in the air, the average SpO_2_ is significantly reduced (De Bels et al. 2019). The air at high altitudes is thinner, approximately 60%–70% of the air density at sea level, with oxygen content 35%–40% lower than at sea level, and atmospheric pressure is only half that at sea level (Lefferts et al. 2020; Savla et al. 2018). The brain is the organ most sensitive to hypoxia. The lower oxygen concentration in high‐altitude environments causes hypoxic damage to the blood vessel walls, leading to vascular dysfunction, increased blood volume, disrupted cerebral circulation, and ultimately contributing to cerebrovascular diseases (Knight‐Greenfield et al. 2019). There is a significant negative correlation between altitude and temperature, meaning that as altitude increases, temperature generally decreases. On average, for every 1000‐m increase in altitude, the temperature drops by about 6.5°C, a phenomenon known as the “lapse rate” (Douglass et al. 2004). Therefore, high‐altitude regions are characterized by the “three lows”—low pressure, low oxygen, and low temperature. Furthermore, high altitude also induces various other physiological adaptations in the body, including but not limited to increased hemoglobin and hematocrit levels, changes in lung volumes, and enhanced standing endurance (Hameed et al. 2024).

In addition to the environmental differences between high‐altitude and low‐altitude regions, there are also anatomical differences in populations living at high altitudes. Droma et al. (1991) found that people in high‐altitude areas have larger chest circumferences compared to those at lower altitudes, which is believed to improve gas exchange efficiency and help better adapt to the hypoxic high‐altitude environment. These changes reflect the evolutionary adaptations of humans, as discussed in Darwin's *On the Origin of Species*, to adjust to their natural environment. It is worth noting that due to the cold, hypoxic conditions in high‐altitude regions and the influence of ethnic dietary habits, people living in high‐altitude areas tend to consume more meat and fewer vegetables. Locals prefer drinking alcohol and eating high‐fat foods such as beef, mutton, and organ meats to stay warm in the cold climate and counteract the low‐calorie, hypoxic environment. As a result, the diet in high‐altitude regions is predominantly high in fat, high in cholesterol, and low in vitamins (Li et al. 2022; M. Liu et al. 2021). These differences have sparked ongoing exploration by numerous scholars into the relatively under‐researched area of high‐altitude populations.

## 4 Ischemic Stroke

Stroke is an acute cerebrovascular disease characterized by vascular causes of neurological dysfunction and focal brain injury in the central nervous system (Sacco et al. 2013). It is classified into IS and HS, with IS being more common than HS (Wu and Liu 2024). Epidemiological data indicate that the annual incidence of stroke exceeds 16 million. Among various cerebrovascular diseases, cerebral infarction accounts for 48.4%, cerebral hemorrhage for 37.2%, subarachnoid hemorrhage (SAH) for 9.8%, and cerebral embolism for 4.6%. Studies have shown that individuals living in high‐altitude areas have a significantly higher prevalence of various cerebrovascular diseases, with IS being the most common (Syed et al. 2022).

Regarding the pathogenesis of IS in high‐altitude regions, a rat model study found that, compared to low‐altitude areas, the expression of NMDAR, CaMKII, and P‐CaMKII proteins was reduced in high‐altitude regions, while the number of red blood cells, hemoglobin, and hematocrit levels were increased. This suggests that the mechanism of stroke in high‐altitude areas is related to decreased expression of NMDAR and CaMKII, as well as an increase in red blood cells and hemoglobin (M. Zhu et al. 2021). Studies indicate that at high altitudes, particularly above 2500 m, the risk of thrombosis and IS is increased (Hameed et al. 2024). IS patients living at high altitudes are predominantly male, younger, with an average age of diagnosis for IS around 40 years (Niaz and Nayyar 2003; Jha et al. 2002; Jaillard et al. 1995), which is 5 years younger than the average age at low altitudes (M. Liu et al. 2021). Additionally, Liu et al. found that IS at high altitudes tend to have larger infarct volumes, and the infarct volume increases with altitude, especially in non‐lacunar infarcts. This suggests that large vessels may be more susceptible. Furthermore, although the NIHSS scores at different altitudes did not show direct differences, the incidence of consciousness disturbance was higher in high‐altitude areas, which indicates that IS in these regions tends to be more severe. M. Liu et al. (2021) therefore suggest that IS occurs earlier and more severely in the Tibetan Plateau region of China.

In high‐altitude areas, IS and its risk factors different from those in low‐altitude regions. The prevalence of diabetes, hyperlipidemia, and coronary artery disease among IS patients is lower in high‐altitude areas. Blood glucose levels in high‐altitude IS patients are associated with the cold climate and the expression levels of hypoxia‐inducible factor (HIF) (Yan et al. 2022). HIF is a transcription factor known to have anti‐ischemic effects and to improve heart function. It senses cellular hypoxia and helps cells adapt and survive in hypoxic environments by upregulating erythropoietin, promoting angiogenesis, and enhancing glycolysis (Mitroshina et al. 2021). Whole‐genome sequencing of populations exposed to chronic hypoxia in high‐altitude areas has revealed high expression of the HIF signaling pathway (Storz 2021). HIF‐1α primarily acts on skeletal muscle, increasing glucose utilization and glycolysis, while HIF‐2α primarily affects the liver, inhibiting hepatic gluconeogenesis. This results in significantly lower fasting blood glucose levels in populations living at high altitudes compared to those at sea level (K. Wei et al. 2013; Woolcott et al. 2015). Additionally, polycythemia, hyperhomocysteinemia, and smoking have a higher prevalence among IS patients in high‐altitude regions (M. Liu et al. 2021), and smoking appears to be more strongly associated with IS in these areas compared to others (Li et al. 2022). The prevalence of hypertension, alcohol consumption, and atrial fibrillation is similar to that in low‐altitude regions. However, Li et al. hold a differing view regarding atrial fibrillation in high‐altitude regions, suggesting that the risk of atrial fibrillation is higher in these areas. This may be related to the higher incidence of pulmonary hypertension and congenital heart disease in hypoxic environments at high altitudes, and atrial fibrillation increases the risk of cardioembolic stroke (Li et al. 2022). The blood pressure levels in high‐altitude regions differ from those in low‐altitude areas. Systolic blood pressure is lower in high‐altitude regions compared to low‐altitude areas, while diastolic blood pressure is higher. There is no difference in mean arterial pressure between the altitude groups. This difference results in a lower pulse pressure in high‐altitude regions (M. Liu et al. 2021). This is primarily attributed to the increased metabolism induced by hypoxia and the rise in heart rate and peripheral resistance due to erythrocytosis (Pappano and Gil Wier 2013). The C‐reactive protein levels in acute IS patients at high altitude are significantly elevated, but no difference is observed between altitudes of 2550 and 4200 m (M. Liu et al. 2021). Additionally, the proportion of patients achieving functional independence (modified Rankin scale, mRS < 2) is significantly lower in high‐altitude IS patients compared to those in low‐altitude areas (Yan et al. 2022).

Currently, there is still controversy regarding the effect of altitude on the incidence of IS. A 2009 ecological study from Switzerland showed that for every 1000‐m increase in altitude, the risk of stroke decreased by 12% (Faeh et al. 2009). One possible explanation for this is the better adaptation of high‐altitude populations to hypoxia, along with the involvement of HIF. However, epidemiological studies from India and Kazakhstan indicate that the risk of IS is significantly higher in high‐altitude areas (Niaz and Nayyar 2003; Jha et al. 2002). One hypothesis suggests that the mortality rate of IS in high‐altitude areas is related to the duration of exposure to high altitude. Acute exposure to high altitude may increase IS mortality, whereas chronic exposure to the high‐altitude environment may reduce IS mortality (Ortiz‐Prado, Cordovez, et al. 2022). Furthermore, individuals who suffer IS in a low‐pressure, low‐oxygen environment tend to have a higher mortality rate, although the brain system damage in survivors may not be as severe (M. Zhu et al. 2021). A retrospective comparative study between Tibet and Beijing revealed that, based on the infarction location, anterior circulation infarction was more common. According to the TOAST classification, the incidence of large‐artery atherosclerosis stroke was higher (Lu et al. 2020).

From the perspective of treatment options, there are also differences between the two regions. The proportion of IS patients receiving intravenous thrombolysis is lower in high‐altitude areas, possibly due to the difficulty of transportation in these regions and the lack of awareness about IS in high‐altitude populations (Lu et al. 2020). As a result, patients often miss the time window for intravenous thrombolysis by the time they reach the hospital, as these areas still adhere to a 6 h from onset deadline for thrombolysis. In the Tibet Autonomous Region, hospitalized patients are more likely to use intracranial pressure‐lowering medications, while in Beijing, anticoagulants are more frequently prescribed. This difference reflects distinct pathophysiological mechanisms of IS between the two regions. In Tibet, IS may be more associated with hemodynamic changes, leading to larger areas of damage and a higher likelihood of edema. In contrast, in Beijing, IS is more related to atherosclerosis and thrombosis, with patients often presenting with more progressive strokes (Liebeskind et al. 2019). The above conclusions are detailed in Table 1.

**TABLE 1:** Research on ischemic stroke in high‐altitude areas.

Author	Publication date	Type	Altitude	Conclusion
Lu et al. (2020)	2020	Retrospective study	Not mentioned	In Tibet, stroke patients are younger, with erythrocytosis and hyperhomocysteinemia as independent risk factors.
M. Zhu et al. (2021)	2021	Animal study	4000 m	Increased blood viscosity is a risk factor and pathophysiological mechanism for the onset of ischemic stroke in high‐altitude regions.
M. Liu et al. (2021)	2021	Retrospective study	≥2550 m	Acute ischemic stroke occurs at an earlier onset and presents more severely in high‐altitude regions of China.
Yan et al. (2022)	2022	Retrospective study	2275 m on average	In anterior circulation acute ischemic stroke, high‐altitude patients present with younger age, larger infarct volume, more severe deficits, and worse prognosis than low‐altitude counterparts.
Ortiz‐Prado, Cordovez, et al. (2022)	2022	Systematic review	≥ 1500 m	An altitude greater than 3500 m increases the risk of ischemic stroke, while an altitude below 3500 m appears to reduce the risk.
Hameed et al. (2024)	2024	Review	Not mentioned	Altitude greater than 3500 m increases the risk of ischemic stroke, whereas populations residing at altitudes between 1500 and 2500 m appear to have a protective effect against stroke.

## 5 Hemorrhagic Stroke

HS refers to bleeding within the brain parenchyma, SAH or ventricular hemorrhage caused by the rupture of cerebral blood vessels. Intracerebral hemorrhage (ICH) is the second most common and most fatal subtype of stroke (van Asch et al. 2010). Research on HS in low‐altitude regions has been abundant, whereas studies on HS in high‐altitude regions remain relatively scarce. Studies have shown that ICH in high‐altitude regions also has a high disability and mortality rate. HS in the Tibet region may be more common due to factors such as hypertension, heavy alcohol consumption, a meat‐based diet, and genetic differences within the population (Fang et al. 2011). An animal study suggests that ICH in high‐altitude regions is more severe than in lowland areas due to the unique low‐pressure, hypoxic environment. This leads to more severe hypertension and edema following the hemorrhage, resulting in poorer neurological recovery. The more severe brain edema caused by hypoxia after the hemorrhage may increase intracranial pressure, ultimately leading to more significant neurological deficits. In the hypoxic environment of high altitudes, the repair of damaged brain tissue and the recovery of neurological function are delayed (H. Zhu et al. 2013). Other studies suggest that the pathogenesis of ICH at high altitudes is due to the low‐pressure hypoxia‐induced damage to the vascular walls. Severe traumatic vessel rupture, caused by factors such as polycythemia, hypertension, heavy alcohol consumption, and head trauma, may lead to ICH (Fang et al. 2011; Zhao et al. 2010). Wei et al. study suggests that hemoglobin concentration significantly affects the mortality and disability rates of hypertension‐related ICH in high‐altitude regions. A linear relationship exists between high hemoglobin concentrations and neurological recovery. When hemoglobin concentration is < 180 g/L, increases in hemoglobin concentration have little effect on neurological recovery after ICH. However, when the concentration > 180 g/L, increases in hemoglobin concentration have a marked impact on neurological function. This phenomenon may be due to the fact that when hemoglobin concentration > 180 g/L, the increase can affect blood flow, leading to cerebral hypoxia and exacerbating cerebral edema (L. Wei et al. 2019). At high altitudes, the red blood cell count increases, and consequently, hemoglobin concentration also rises (Jha et al. 2002; Grotta et al. 1982). Additionally, hemoglobin itself can cause damage to brain tissue following a HS (L. Wei et al. 2019). The unique dietary habits of high‐altitude populations also constitute a risk factor for HS. The diet in these regions typically consists of high‐fat, high‐salt foods, which are recognized risk factors for HS (Foroughi et al. 2013; He et al. 2003). Chen et al. found that, compared to Han Chinese patients with ICH, Tibetan patients from high‐altitude regions exhibited some distinct characteristics in baseline measurements, comorbidity rates, hematoma location, in‐hospital complication risks, and clinical outcomes. Even after adjusting for factors such as age, gender, overweight status, hemoglobin concentration, smoking, hypertension history, diabetes history, previous HS, brainstem hemorrhage, hematoma expansion complications, and cerebral infarction, the Tibetan group showed a significantly higher risk of poor outcomes associated with ICH (RR = 2.45, 95% CI 1.39–4.33). This suggests the presence of ethnic differences in HS outcomes at high altitudes (Chen et al. 2018).

Aneurysms, as one of the causes of SAH, have been shown to pose a higher risk of occurrence and poorer prognosis in Tibetan patients with blister‐like aneurysms (BLAs) compared to Han Chinese patients. These patients also experience a higher incidence of HS. Endovascular treatment has been found to provide greater benefits in terms of reducing cerebral infarction and improving neurological recovery outcomes for Tibetan patients with BLAs. Therefore, for this group of patients, endovascular therapy may be a safer and more effective treatment option compared to surgical intervention. However, studies have shown that patients with neurogenic SAH who undergo endovascular treatment have a higher recurrence rate, and the long‐term prognosis remains unclear (Chen et al. 2017; Uda et al. 2001). Studies have shown that there are differences in the common locations of BLAs between Tibetan and Han populations. In the Han group, the location of aneurysmal lesions appeared to be relatively consistent. Among 34 Han patients, 33 had BLAs at the typical location on the dorsal wall of the internal carotid artery, while 1 patient had an aneurysmal lesion at an atypical location on the middle cerebral artery. In the Tibetan group, among 19 patients with BLAs, 14 had aneurysms at the typical location on the dorsal wall of the internal carotid artery, which does not branch. The remaining five patients had aneurysms at atypical sites: one in the anterior cerebral artery, two in the posterior communicating artery, one in the middle cerebral artery, and one in the C3 segment of the common carotid artery. Atypical aneurysmal lesions were notably more common in the Tibetan group than in the Han group. Furthermore, at various follow‐up time points, the mortality rate in the Tibetan group was higher than that in the Han group (Chen et al. 2017).

In terms of treatment, craniectomy and hematoma aspiration with drainage can effectively reduce the rapidly increasing intracranial pressure due to ICH, thereby protecting the patient's neurological function and reducing the incidence of brain herniation. The time from admission to surgery is associated with the patient's neurological outcome: the longer the time from admission to surgery, the worse the prognosis (L. J. Wei et al. 2021). For ICH patients in high‐altitude areas, Luo et al. (2023) recommend actively monitoring hemoglobin levels, controlling blood pressure, and improving tissue oxygenation, which will contribute to better patient outcomes. Additionally, an animal study suggests that hyperbaric oxygen therapy can improve neurological deficits following ICH in pigs at high altitudes (H. Zhu et al. 2015). The detailed sources for the above conclusions are summarized in Table 2.

**TABLE 2:** Research on hemorrhagic stroke in high‐altitude areas.

Author	Publication date	Type	Altitude	Conclusion
H. Zhu et al. (2013)	2013	Animal study	4000 m	The damage caused by intracerebral hemorrhage is more severe in high‐altitude areas than in lowland regions.
H. Zhu et al. (2015)	2015	Animal study	Not mentioned	Early hyperbaric oxygen therapy can alleviate acute brain injury following intracerebral hemorrhage in high‐altitude areas.
Chen et al. (2017)	2017	Retrospective study	4500 m	Tibetan patients have a higher risk of blister‐like aneurysms, atypical lesions, higher infarction rates, and worse prognosis, but benefit more from endovascular treatment.
Chen et al. (2018)	2018	Retrospective study	4500 m	Tibetan patients with spontaneous intracerebral hemorrhage in high‐altitude regions exhibit unique characteristics compared to Han Chinese patients.
L. Wei et al. (2019)	2019	Retrospective study	≥ 1500 m	Hemoglobin concentration affects the mortality and disability rates of hypertensive intracerebral hemorrhage in high‐altitude areas.
L. J. Wei et al. (2021)	2021	Retrospective study	≥ 1500 m	Craniectomy and hematoma puncture drainage are effective methods for treating hypertensive intracerebral hemorrhage.
Luo et al. (2023)	2023	Letter	Not mentioned	The prognosis of intracerebral hemorrhage is worse in high‐altitude areas.

## 6 Post‐stroke Cognitive Impairment

Cognitive impairment refers to functional deficits in one or more cognitive domains, including memory, calculation, orientation, attention, language, executive function, reasoning, and visuospatial abilities. PSCI refers to the decline in cognitive function that occurs following a stroke, encompassing both mild cognitive impairment and dementia, although there is no universally accepted definition. PSCI is one of the most common neurological sequelae following a stroke (Kim et al. 2022). In China, the prevalence of PSCI among stroke survivors is nearly 81% (Qu et al. 2015). PSCI negatively impacts quality of life and is associated with poor functional outcomes after a stroke (Yuan et al. 2021; Liao et al. 2022). Currently, there is a considerable amount of research on cognitive impairment in high‐altitude regions (Ji et al. 2021; Ma et al. 2024; J. Liu et al. 2020). However, as of January 13, 2025, only one multicenter cross‐sectional study has been found regarding PSCI in high‐altitude areas (Li et al. 2023). The study found that the incidence of PSCI in the Qinghai region was 47.8%, which contrasts sharply with the 81% incidence previously observed in low‐altitude areas. People in high‐altitude regions experience long‐term hypoxia, which affects both physiological and psychological functions in complex ways (Davis and Hackett 2017), including cognitive function and mood (Dykiert et al. 2010; Heinrich et al. 2019). Study has shown that immigrants born in low‐altitude areas are less likely to develop cognitive impairment compared to the native residents of Qinghai. In addition, the study found that early PSCI was negatively correlated with educational level, nonimmigrant status (born in low‐altitude areas), consumption of fruits, beef, and lamb, Activities of Daily Living scores, and leisure activities. These factors were found to have a protective effect on PSCI. On the other hand, older age, female gender, and consumption of yak butter were identified as risk factors (Li et al. 2023). We believe that this multicenter cross‐sectional study lays a solid foundation for future research on PSCI in high‐altitude regions.

## 7 Flight‐Associated Stroke

With the advancement of society and technology, travel methods have become more diverse. Air travel has now become commonplace, with statistics showing that nearly 2 billion people travel by air each year (Humaidan et al. 2016). Flight‐associated stroke (FAS) is relatively rare. Aircraft typically fly at an average altitude of 9000–12,000 m, but the cabin pressure is only adjusted to approximately 552–632 mmHg, which is equivalent to an altitude of 1500–2500 m above sea level (Hameed et al. 2024; Medical Aspects of Transportation aboard Commercial Aircraft. AMA Commission on Emergency Medical Services 1982). Therefore, FAS can be considered a variant of high‐altitude stroke. Therefore, FAS can be considered a variant of high‐altitude stroke. According to Dalton's law, in this scenario, the absolute partial pressure of oxygen decreases, while its proportion in the air composition remains unchanged. This leads to a reduction in hemoglobin saturation for all air passengers, with SpO₂ dropping to 85%–91% of normal values. Studies have found that 67% of strokes related to severe carotid artery stenosis or occlusion occurred during longer flights (> 4 h) (Gradwell and Risdall 2005; Humphreys et al. 2005). Additionally, Cui et al. (2014) found that conditions aboard commercial aircraft may increase the likelihood of intracranial aneurysm rupture, leading to SAH. A study suggests that early air transport for patients with nontraumatic ICH is considered safe and effective, facilitating early diagnosis and shortening the time to surgical intervention, patients did not experience significant clinical deterioration during transport, however, blood pressure should be carefully monitored during transit. For safety reasons, air transport should ideally be scheduled during the daytime (Cavalcante and Ormond 2018). Another case report highlights that for patients with intracranial space‐occupying lesions, caution should be exercised regarding air travel, even if air travel is chosen, prophylactic medication should be administered (Mahdavi et al. 2010). As airplanes become an increasingly common mode of travel, with more people engaging in air travel, the safety of air passengers should be better ensured. FAS provide valuable insights for the future development of aviation medicine and may become a significant area of focus in stroke research, requiring further investigation and exploration.

## 8 Preventive Measures for Stroke

Stroke remains one of the leading causes of death and disability worldwide (Soleimanpour 2023) and is the second most common cause of mortality globally (Nikbakht et al. 2022). In middle‐income countries, cerebrovascular disease ranks as the primary cause of death (Ayromlou et al. 2014). According to data from the GBD 2019 Stroke Collaborators, the prevalence of stroke in China is 1.5 times higher than the global average (2.0% vs.1.3%) (GBD 2019 Stroke Collaborators 2021). Tu et al. (2023) reported that in China, the prevalence of stroke at different altitudes ranged from 0.29% to 11.90%, the incidence rate from 35.69/100,000 to 3446.67/100,000, and the mortality rate from 17.36/100,000 to 2616.45/100,000. However, the definite association between high‐altitude exposure and stroke burden has not yet been well‐established. Interestingly, another study indicated that physical inactivity is associated with an increased risk of stroke (Tu et al. 2024). Additional research has shown that populations living at high altitudes tend to have diets high in cholesterol and fat but low in vitamins, with a higher prevalence of smoking and alcohol consumption. Moreover, the association between smoking and stroke appears to be stronger at higher elevations (Li et al. 2022; M. Liu et al. 2021).

Currently, medical resources in high‐altitude regions are significantly scarcer compared to lowland areas. Although intravenous thrombolysis remains an effective treatment for IS, its utilization rate among high‐altitude populations is notably low (Lu et al. 2020). Ayromlou et al. (2013) reasonably attributed this phenomenon primarily to poor public recognition of stroke symptoms and the lack of rapid transportation to hospitals. In China, some hospitals in remote regions are still unable to provide thrombolytic therapy. Notably, there is a scarcity of research examining the impact of ethnic differences on stroke risk in high‐altitude populations, warranting further investigation. Therefore, the development of targeted stroke prevention strategies for high‐altitude regions is urgently needed. The following recommendations may be considered for the prevention of stroke in high‐altitude regions: (1) A low‐fat diet should be promoted, with an emphasis on consuming fresh fruits and vegetables. In addition, smoking and alcohol consumption must be strictly avoided, while regular physical exercise should be encouraged to enhance physical fitness and reduce the risk of stroke. (2) Public health authorities should further strengthen the development of high‐altitude stroke centers and establish specialized high‐altitude laboratories to improve the diagnosis and research capabilities for stroke‐related diseases in high‐altitude regions. Additionally, public health education on stroke should be enhanced for high‐altitude populations to raise awareness and reduce treatment delays due to insufficient knowledge. A bilingual system for health education and scientific dissemination should be established to effectively address communication barriers arising from linguistic differences, preventing negative impacts on disease diagnosis and treatment. (3) Further exploration is needed to assess the impact of ethnic cultural practices and genetic predispositions on the occurrence and prognosis of stroke in high‐altitude regions, providing scientific evidence for targeted stroke prevention strategies.

As the number of air travelers continues to rise, the importance of aeromedical services is increasingly emphasized. To address the specific issue of FAS, the following recommendations may be considered: (1) Comprehensive assessments, particularly for stroke patients, should be conducted before flight to reduce the incidence of adverse events during air transport. (2) For patients with intracranial space‐occupying lesions or intracranial aneurysms, air travel should be approached with caution. If air travel is unavoidable, prophylactic medication should be considered in advance, such as corticosteroids, acetazolamide, and supplemental oxygen (Mahdavi et al. 2010). (3) Acetazolamide, ibuprofen, and dexamethasone have been confirmed to have positive effects in acute high‐altitude exposure (Gatterer et al. 2024). Given the environmental similarities between flight‐related stroke and high‐altitude exposure, these medications may also have potential value in the prevention and treatment of flight‐related stroke. However, their specific effects require further research to confirm.

We hope these recommendations will contribute to the development of future prevention strategies for stroke patients living at high altitudes.

## 9 Limitations of the Study

Based on the summary above, we identify several significant gaps in stroke research in high‐altitude areas, which are summarized as follows: (1) There is a lack of research on endovascular treatment for IS in high‐altitude regions, and the efficacy of endovascular treatment for IS patients in these areas remains to be explored. (2) Study of Lu et al. (2020) only mentioned the intravenous thrombolysis rate, without exploring its efficacy in IS patients in high‐altitude regions. (3) Study of H. Zhu et al. (2015) suggests that hyperbaric oxygen therapy can improve neurological function in patients with ICH in high‐altitude regions, but its efficacy in patients with IS at high altitudes remains to be explored. (4) The research on PSCI in high‐altitude regions is limited to a single multicenter cross‐sectional study (Li et al. 2023), and further high‐level studies are needed. To further validate our hypothesis, we conducted a relevant research search in the Chinese Clinical Trial Registry (ChiCTR) and ClinicalTrials.gov. Using the search query (plateau OR high altitude OR high‐altitude) AND (stroke OR cerebral infarct* OR cerebral hemo* OR Apoplexy) with a cutoff date of January 13, 2025, we identified three relevant studies in the ChiCTR: ChiCTR2400092762, ChiCTR2400090500, and ChiCTR2400087157. Among them, ChiCTR2400092762 is a study conducted by us that explores the efficacy of endovascular treatment. ChiCTR2400090500 investigates the effects of hyperbaric oxygen therapy following intravenous thrombolysis, while ChiCTR2400087157 explores the clinical characteristics and prevention strategies for cerebral infarction in high‐altitude regions. We searched Clinical Trials and found 26 results. After reviewing each one, none of them met the criteria. Therefore, there are still significant gaps in the research related to stroke in high‐altitude areas.

## 10 Conclusion and Outlook

Through a literature review, we explored the current pathophysiology, risk factors, and epidemiological trends of stroke in high‐altitude regions, and highlighted the existing gaps in the field of stroke research in these areas. In addition, we conducted a search on clinical trial registration platforms to identify feasible research directions for stroke in high‐altitude regions, aiming to provide valuable insights for future research and practice in high‐altitude stroke.

## Author Contributions

**Chuanxi Duan**: writing – original draft. **Lijun Wu**: writing – original draft. **Yuding Luo**: writing – original draft. **Jiali Zhang**: investigation. **Pingchuan Liu**: investigation. **Fanzhou Ren**: investigation. **Junhao Li**: investigation. **Hai Xiong**: investigation. **Jian Wang**: conceptualization, writing – review and editing.

## Conflicts of Interest

The authors declare no conflicts of interest.

## Peer Review

The peer review history for this article is available at https://publons.com/publon/10.1002/brb3.70626.

## Data Availability

Data sharing is not applicable to this article, as no new data were created or analyzed in this study.
